# Interactive processing and visualization of image data for biomedical and life science applications

**DOI:** 10.1186/1471-2121-8-S1-S10

**Published:** 2007-07-10

**Authors:** Oliver G Staadt, Vijay Natarajan, Gunther H Weber, David F Wiley, Bernd Hamann

**Affiliations:** 1 Institute for Data Analysis and Visualization and Department of Computer Science, University of California, Davis, CA, USA; 2 Department of Computer Science and Automation, Indian Institute of Science, Bangalore, India; 3 Computational Research Division, Lawrence Berkeley National Laboratory, California, USA; 4 Stratovan Corporation, Woodland, CA, USA

## Abstract

**Background:**

Applications in biomedical science and life science produce large data sets using increasingly powerful imaging devices and computer simulations. It is becoming increasingly difficult for scientists to explore and analyze these data using traditional tools. Interactive data processing and visualization tools can support scientists to overcome these limitations.

**Results:**

We show that new data processing tools and visualization systems can be used successfully in biomedical and life science applications. We present an adaptive high-resolution display system suitable for biomedical image data, algorithms for analyzing and visualization protein surfaces and retinal optical coherence tomography data, and visualization tools for 3D gene expression data.

**Conclusion:**

We demonstrated that interactive processing and visualization methods and systems can support scientists in a variety of biomedical and life science application areas concerned with massive data analysis.

## Background

Recent advances in imaging technology have led to a rapid increase in the size and complexity of biological image data sets. It is often not feasible to explore massive biological image data with conventional analysis and visualization tools. Interactive visualization techniques can support life scientists in the exploration and analysis of biological image data.

Conventional visualization methods often require significant processing time, which limits high-throughput analysis. Interactive visualization systems maintain a closed loop between the user and the system and, thus, need to be very fast. Building such a system requires the development of new visualization methods, and there exists also need to design new and effective interaction techniques.

We provide an overview of selected methods in high-resolution display technology and visualization techniques developed at the Institute for Data Analysis and Visualization (IDAV) at UC Davis, in close collaboration with scientists from other institutions.

### High-resolution display technology for exploration of biomedical image data

Large display environments have become increasingly important over the past decade and are used frequently for displaying high-resolution data resulting from imaging applications and computer simulations. The size and complexity of biological and biomedical data sets increases steadily, and the resolution of single-projector displays is no longer sufficient to reveal details without zooming in and, thus, loosing important context information. One possible solution to this problem is the use of tiled displays that use multiple projectors to increase the total resolution of the system. Even though high-quality projectors are now available at reasonable cost, increasing the number of tiles by adding more rows and columns increases the cost of the system significantly. For example, adding one row and one column to a four-by-three-tile display increases the number of projectors (and rendering nodes) from 12 to 20.

It can be argued that system resolution should be increased homogeneously across the display area. For example, it is often not necessary to increase the resolution in the periphery of the display by the same amount as in the center of the display. One way of exploiting this is to have a higher-resolution region in the center of the lower-resolution display. However, a fixed-location inset constrains user interaction. The human visual system overcomes the problem of a static foveal region in the retina with saccades, rapid movement of the eye between fixation points.

**Figure 1:**
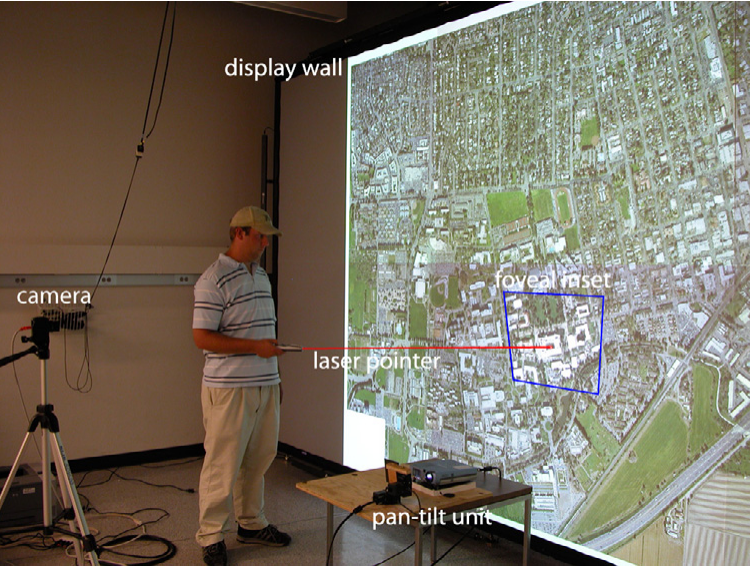
**Foveal inset**. The user directs the projection of the foveal inset using a laser pointer. The size of the projected inset is significantly smaller than the tiles of the rear-projected display wall, thus providing a higher resolution.

**Figure 2:**
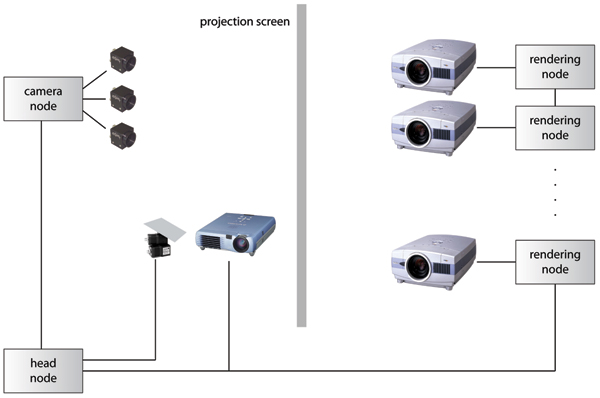
**Foveal inset system illustration**. Illustration of the foveal inset display system configuration including display screen, projectors, cameras, and pan-tilt unit. The *head node *generates the projection of the foveal inset, controls the PTU position, and controls the *rendering nodes *and the *camera nodes*. Each rendering node projects one tile onto the rear of the projection screen. The camera nodes process video data for calibration and laser pointer-based interaction. The foveal inset is front-projected onto the projection screen.

We have developed a positional foveal inset mechanism for tiled displays [1], a novel mechanism for interacting with large displays. A high-resolution projector and a mirror mounted on a pan-tilt unit (PTU) are used to move the foveal inset on a tiled display. This method supports examination of areas of interest in high detail without the expense of adding more tiles to the display. It also allows the system to keep pace with advancements in projector technology. Instead of upgrading a large number of projectors, only a single foveal inset projector needs to be replaced. Figure 1 shows a user directing interactively the location of the foveal inset using a laser pointer and Figure 2 illustrates the components of the prototype system.

### Analysis and visualization of protein surfaces

It is widely believed that the structure of a macromolecule determines its function [2]. Structural analysis of a macromolecule is a challenging problem that requires development of fast and high-quality imaging technology, efficient schemes to represent 3D protein surface structure, good understanding of the interplay between geometry and the physio-chemical properties of the molecule, and efficient tools that support interactive visualization of the macromolecule. We focus on proteins, an important subclass of macromolecules.

The 3D structure of a protein can be represented in several ways: as a union of balls, a collection of atom locations in 3D space, isosurfaces of a 3D potential fields, or as a smooth molecular surface. We use a surface representation, in particular the *skin surface *[3], which consists of a finite collection of quadric patches that together form a smooth surface. Efficient algorithms [4] have been developed to construct a triangle mesh representation of the skin surface given the atom locations and radii as specified in the protein data bank. We prefer the skin surface over surface representations including the *Van der Waal surface *[5] and the *molecular surface *[6], because the latter surfaces suffer from artifacts due to discontinuities and self-intersections, respectively.

### Visualization tools for 3D gene expression data

In recent years, the genomes of an increasing number of species have been sequenced and published. While we are able to decipher the locations of the protein coding portions of many genes from genomic sequence information, the mechanisms that control when and where a gene is expressed are still poorly understood. The Berkeley *Drosophila *Transcription Network Project (BDTNP) is a multidisciplinary effort, including UC Davis scientists, whose goal is to decipher how the regulatory information contained in DNA sequences directs the patterns of gene expression underlying animal development. Using the early embryo of the fruitfly *Drosophila melanogaster *as a model, the BDTNP is developing experimental and computational methods to to describe and analyze 3D gene expression patterns. One aim is to produce an atlas of expression patterns of a large number of genes at cellular resolution and to use this information in combination with other data sets to learn how to understand the control mechanisms in genomic DNA and model the regulatory network.

To construct this atlas, the BDTNP scientists have developed a data processing pipeline to determine the location and extent of nuclei in embryos and measure the relative levels of mRNA expression in the cytoplasmic regions surrounding each nucleus [7,8]. Embryos are fluorescently stained to detect cell nuclei and expression patterns of selected genes are imaged. Per embryo, the resulting volumetric data sets consist of a stack of 120–140 image slices of 1024-by-1204 pixels each. Fluorescence intensities for the nuclear stain and the fluorescently labeled gene products are captured as separate scalar values per voxel. The obtained images are processed to detect all blastoderm nuclei and compute a segmentation mask for the embryo. This segmentation mask is subsequently used to measure the fluorescent intensities associated with each gene product in the nucleus and in apical and basal parts of the nearby cytoplasm. We generally refer to the measured fluorescent intensities as gene expression levels, assuming that the two are closely correlated, see Luengo et al. [7]. A *Single PointCloud *file that results from this step contains information about the spatial location of each nucleus in an embryo, the nuclear and cellular volumes, and the relative concentrations of gene products (mRNA or protein) associated with each nucleus and cell [7,9,10]. As part of this project, IDAV researchers are developing methods to visualize all types of data encountered in the project ranging from raw image data collected by confocal microscopy imaging to derived PointCloud data. Specific tools permit (i) visualization of raw confocal microscopy data, (ii) interactive control and verification of the image processing algorithms [11], and (iii) visual analysis of complex gene expression patterns in derived PointCloud data [12].

### Visualization and analysis of retinal optical coherence tomography

The leading cause of blindness in the industrialized world is that of age-related macular degeneration (AMD) affecting one in twenty people over the age of sixty [13]. Blindness due to AMD is caused by gradual photoreceptor degradation and eventual cell death over time. An early sign of AMD is the formation of bumpy extracellular deposits called *drusen *between the *retinal pigmented epithelium *(RPE) layer and the *Bruch's membrane*. Identification of drusen can provide and early warning to the onset of AMD allowing better drug treatment in order to slow the progression.

The second leading cause for blindness is glaucoma [14], characterized by the progressive loss of optic nerve axons resulting in a gradual loss of visual field. This begins in the peripheral and slowly imposes tunnel vision upon the patient. This loss is gradual and often goes unnoticed by the patient until it has progressed to a point that treatment is of little help.

We have developed an imaging system based on capturing image volumes using optical coherence tomography (OCT) imaging technology. We subsequently interactively visualize and analyze captured retinal OCT data for the purpose of diagnosing and devising treatment plans. This is accomplished by isolating pathologic features within the retinal volumes, such as retinal layers, for subsequent thickness analysis and comparison to normal and diseased eyes.

## Results and discussion

### High-resolution display technology for exploration of biomedical image data

When the foveal inset is projected onto the display screen, it appears skewed due to the oblique projection used to project onto the display plane. For the foveal inset image to appear aligned with the rest of the display, a homography matrix must be computed to map the foveal inset image plane to the display image plane. The foveal inset projection must be pre-warped using the homography matrix. Additionally, the area in the main display where the foveal inset lies must be removed to avoid image blurring caused by the overlapping tiled display and foveal inset.

#### Calibration

Our system performs a coordinate mapping from the foveal inset image plane to the display image plane. Mapping a point in homogeneous coordinates from one plane to another can be achieved using a three-by-three homogeneous matrix [15]. The mirror which reflects the foveal inset is in a number of different positions as the PTU moves, effectively changing the image plane of the inset. For this reason, a different homography is required for each PTU position. Due to the PTU's high resolution, pre-computing these homographies for each possible pan-tilt angle pair is impractical. Instead, our system calibrates for a configured subset of the possible positions. This allows the range of foveal inset positions to be configured in such a way that all desired areas of the display are covered and minimizes the amount of calibration time and system memory needed to use the system.

#### Interaction

A laser pointer is used to interact with our system [16]. The user can direct the laser pointer to an arbitrary location on the screen and the foveal inset is directed to that location when the laser pointer button is released. More specifically, the center points of each calibrated foveal inset position are used to determine which of the positions is most appropriate for the selected location. When the client is started and has read the calibration data, the center of each foveal inset is projected into the display image plane and inserted into a nearest-neighbor search structure. When the inset controller needs to position the foveal inset, it uses this search structure to find the closest center point to the laser pointer position. The PTU position is set to the corresponding pan-tilt angles and the corresponding homography is used to pre-warp the foveal inset image.

The controller receives messages indicating both the laser pointer position and when the laser pointer is no longer visible. When a positional message is received, the coordinates are internally recorded. When the laser pointer is no longer visible (i. e., when it has been turned off) the client moves the PTU and applies the pre-warp matrix.

#### Experimental results

**Figure 3:**
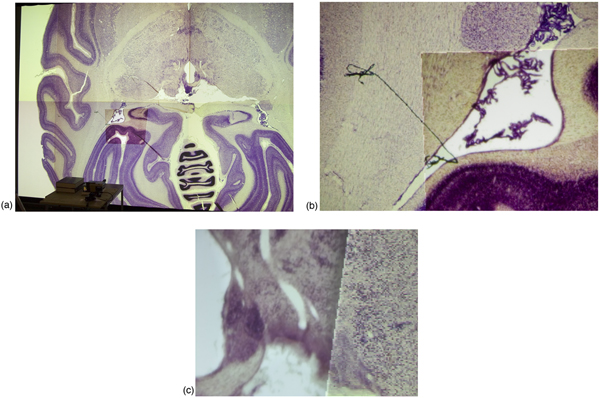
**Visualization of high-resolution slice of cryosection of monkey brain**. a: The foveal inset is projected onto the lower-left tile of the display wall. b-c: Magnified view of the boundary between the inset and the display wall. The foveal inset provides the user with a higher-resolution view of the selected area. c: Magnified view of a high-resolution slice of a cryosection of a monkey brain. The boundary between the high-resolution foveal inset (left) and the lower-resolution display wall (right) is clearly visible. Note that the pixel dimensions of the foveal inset projector and the display wall projector are identical.

To evaluate the impact of higher-resolution foveal insets on applications visualizing high-resolution data, we used a prototype image viewer application. This application, shown in Figure 3, allows a user to interactively pan and zoom very large image files using out-of-core rendering methods. The application uses a quadtree-based multiresolution representation which is created in a pre-processing step, and uses OpenGL to render an image as a set of texture-mapped square tiles at different levels of resolution. The example image shown in the figures is a stained slice from a cryosection of a monkey brain. The image has a resolution of 5000 × 5800 pixels, and the multiresolution representation occupies 113 MB on disk. Since the foveal inset provides a locally increased resolution, and the appropriate level-of-detail for image rendering is chosen based on pixel size, the application automatically renders the image at a higher resolution in the area covered by the inset, see Figure 3b.

### Analysis and visualization of protein surfaces

We have developed a method for segmenting a protein surface into feature defining regions. Each region represents a cavity or a protrusion on the protein surface [17]. The segmentation is computed hierarchically at multiple levels of resolution, thereby identifying features at different scales. This segmentation is useful for visual analysis of the protein and helps in the study of potential active sites of interaction with other proteins.

We assume that the protein surface is given as a triangle mesh in 3D Euclidean space along with a real-valued function *f *defined at each mesh vertex and linearly interpolated within the triangles. We use ideas from Morse theory [18] to related critical points of *f *with shape characteristics of the surface. The *atomic density function *defined by Mitchell et al. [19] identifies the concavity and convexity of points on the surface. We use a variant of the atomic density function to approximate the mean curvature at points on the surface [20].

**Figure 4:**
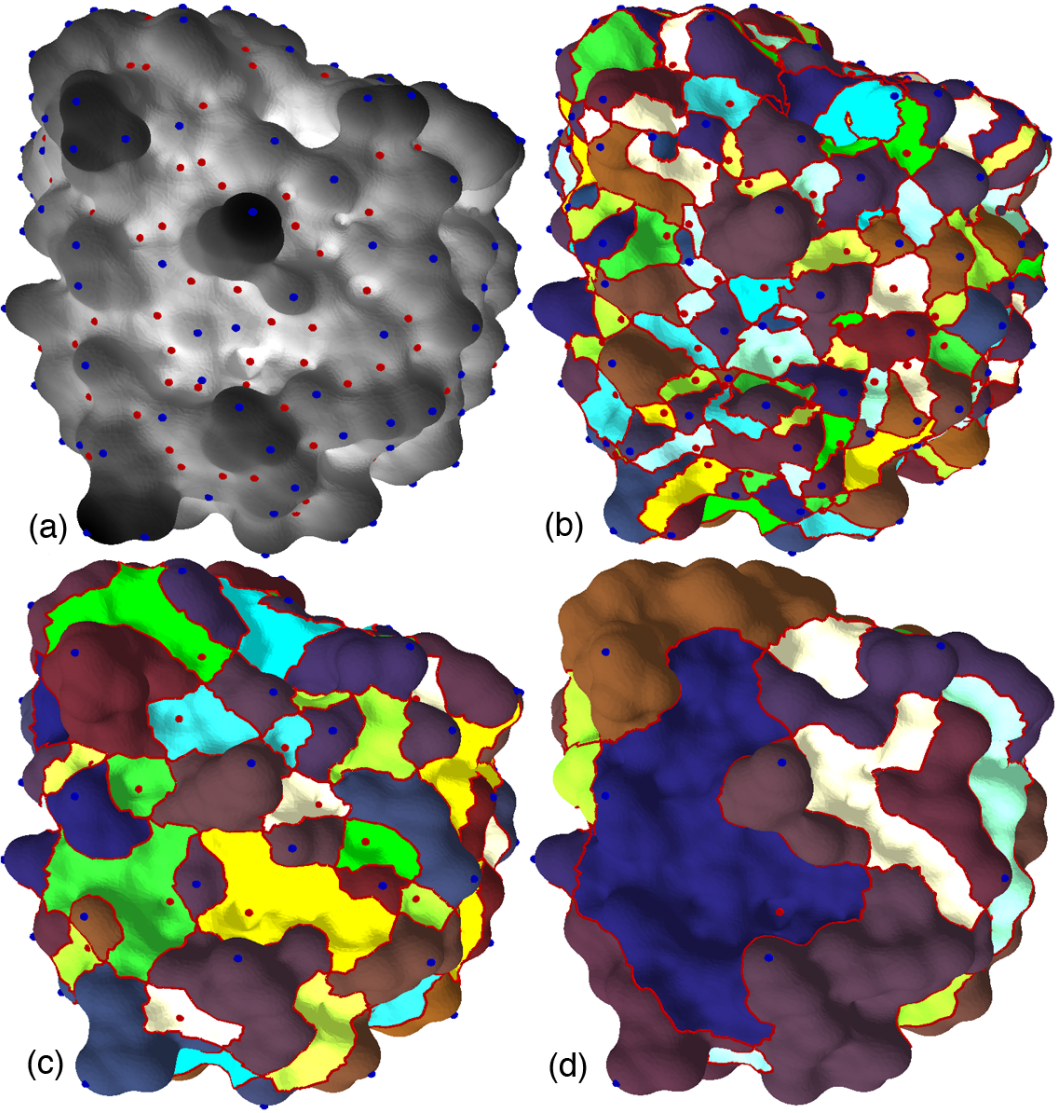
**The atomic density function**. a: Darker regions correspond to protrusions and lighter regions correspond to cavities. b-d: Hierarchical segmentation provides explicit representation of cavities and protrusions at multiple scales.

Figure 4 shows the atomic density function distribution over the surface of a single chain in the protein complex *Barnase-Barstar *(pdb id 1BRS). We are able to extract protrusions and cavities on the surface at multiple scales by applying our segmentation algorithm on this surface.

### Visualization tools for 3D gene expression data

#### Volume rendering of raw confocal microscopy data

**Figure 5:**
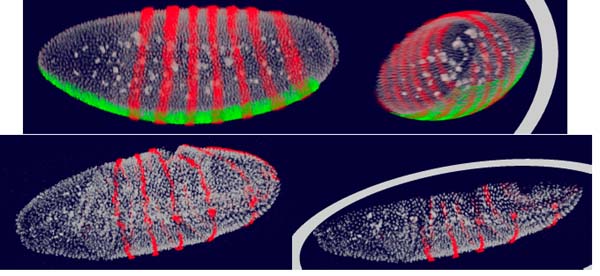
**Visualization of raw confical microscopy images of Drosophila embryo**. The channel stained for DNA is rendered in white. The two (upper panels) or one (lower panels) channels containing information about gene expression are rendered in red and green or red. Utilizing a clipping plane (right panels) it is possible to reveal the embryo interior.

Confocal microscopy images acquired by the BDTNP typically consist of three channels, one channel that is stained for DNA using SytoxGreen and two channels that are stained for gene expression (e.g., *ftz *and *sna*). By adapting novel volume rendering methods [21] to multi-channel confocal microscopy data, every single channel of an image stack is rendered independently by mapping brightness information to color and transparency information. Resulting colors are blended using user-specified weights, supporting seamless blending between channels, including the possibility to show all three channels simultaneously. Using modern PC graphics boards, it is possible to generate volume-rendered images at interactive frame rates [22]. Our volume renderer also supports slicing and rotation tools to make it possible to visualize an arbitrary cross section from an image stack, and to explore the interior of an embryo, see Figure 5.

#### Visualization of segmentation results

**Figure 6:**
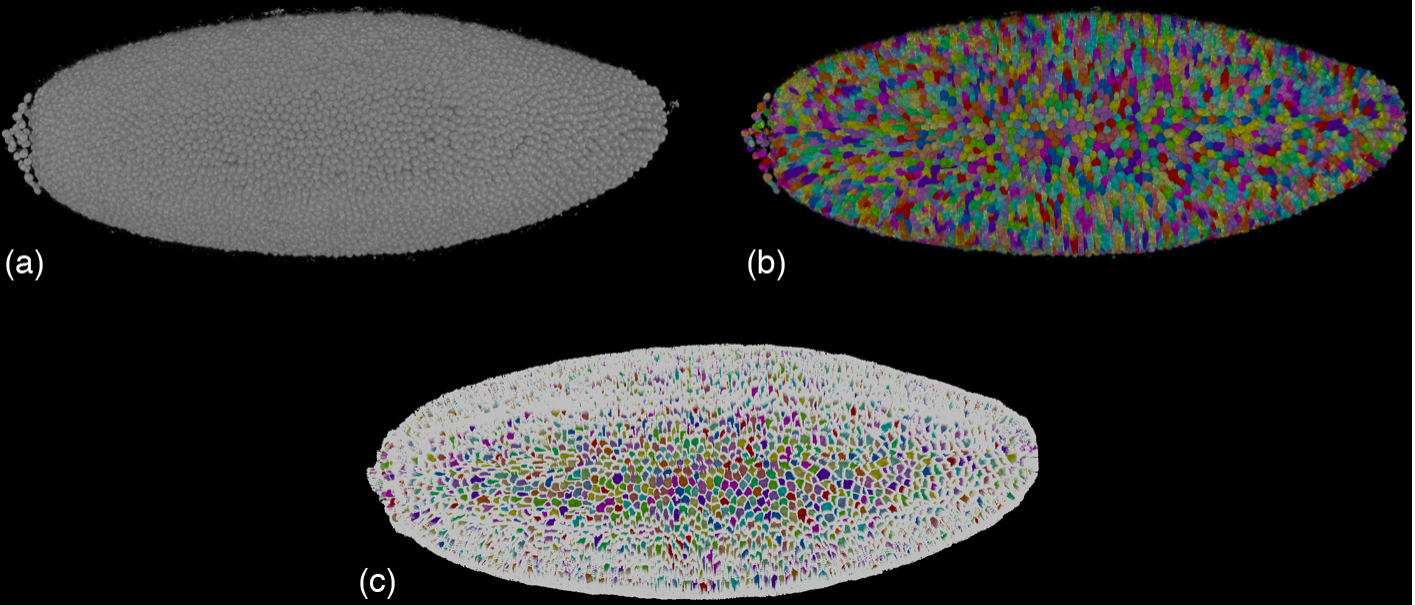
**Rendering DNA channel of Drosophila embryo**. a: Using a gray-scale transfer function. b: Display segmentation information. c: To further emphasize the segmenation, boundaries between individual segmented regions can be added by a fragment program.

We have combined our volume visualization prototype with user interaction tools to support quantitative determination of the accuracy of our nuclear segmentation methods [11]. Using a novel prototype GPU-based volume renderer for segmented volume data, nuclei are rendered by incorporating information obtained from a nuclear segmentation mask. Each nucleus is colored differently and rendered with brightness and opacity that is determined by the original image, utilizing graphics fragment programs. Interactive selection of individual nuclei is made possible by intelligently querying the frame buffer. Our visualization tool provides an interface to MATLAB, making it possible to interact with the segmentation algorithms. This work enabled significant improvements of segmentation accuracy, see Figure 6.

#### Visualization of PointCloud data

Final 3D gene expression data is eventually represented in the form of matrices or PointCloud data that describe nuclear positions and their associated gene expression levels. To decipher regulatory relationships, two aspects of gene expression are of primary interest: (i) gene expression patterns defined by the spatial locations of cells expressing a gene, and (ii) relationships between the expression levels of multiple genes. We have developed a visualization tool [12,23] that utilizes simple but powerful basic principles to take these aspects into account. Multiple views make it possible to show different data aspects without being overwhelmed by the high dimensionality of PointCloud data. Each view emphasizes different data properties, and the interplay between all views makes detailed data analysis possible.

### Visualization and analysis of retinal optical coherence tomography

In order to accurately diagnose, study, and treat retinal diseases such as AMD and glaucoma, researchers have begun capturing volumetric image data using OCT. OCT directs a ray of coherent light produced from a superluminescent diode into a subject's eye wherein the back-reflected light is detected and processed to form an *A-scan*. An A-scan resolves material interfaces of the retinal layers along the ray to an approximate depth of 4.5 *μm*. Diverting the light direction by servo-controlled mirrors allows one to rapidly change the imaging location. Quickly redirecting along a line approximately 8 *mm *in length produces a series of A-scans that, when combined together, form a 2D B-scan similar to a single slice obtained from magnetic resonance imaging (MRI). Rapidly redirecting the imaging plane laterally allows the collection of a series of B-scans resulting in a stack of images that form a image volume roughly 4 *μm *× 8 *mm *× 8 *mm*. It takes several seconds to capture between 200 and 500 B-scans for and entire volume. During this time, normal uncontrollable movement occurs in the patient's eye resulting in an unregistered stack of B-scan images. The individual B-scans are captured quickly enough to have minimal misalignment per scan; however, it is nearly impossible to capture a perfectly registered set of B-scans from any patient due to these movements.

**Figure 7:**
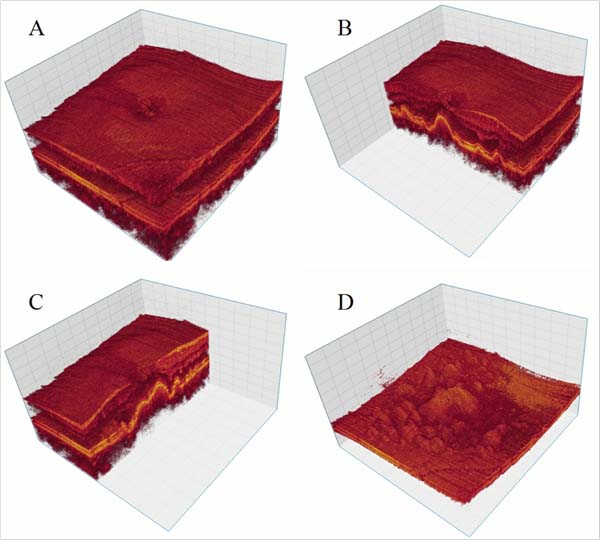
**3D visualization of volumetric retinal OCT data**. 3D visualization of volumetric retinal OCT data. Image A shows the Whole retinal volume. Image B shows the left part of the volume removed by XZ clipping plane. Image C shows the left part of the volume removed by YZ clipping plane. Image D shows a segmented region of the volume.

**Figure 8:**
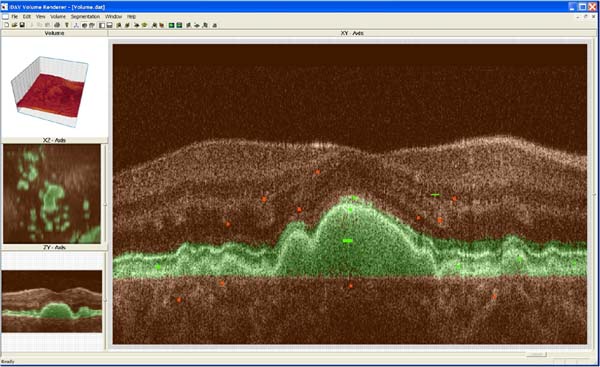
**User interface with SVM training data shown on B-scan**. User interface with SVM training data shown on the B-scan. For training, green and red marks are interactively painted to specify regions of interest and disinterest, respectively. Automated volume segmentation is rendered with a green tint on the 2D cross sections. The isolated region of interest is shown in the upper-left window panel. For this example, the RPE and photoreceptor layers are segmented.

We help ophthalmologists explore the retinal volumes by providing visualization and analysis methods. The visualization techniques are standard hardware-accelerated volume rendering methods, similar to those in [24] see Figure 7, in conjunction with a support vector machine (SVM)-based segmentation method for extracting retinal layers and other structures. The user interface is painting-based so that users paint regions they desire green and undesirable regions red, see [25] see Figure 8. The SVM then isolates the selected region for further processing. These structures are typically rendered as thickness maps, a standard evaluation form, so that practitioners can diagnose and evaluate disease progression.

Over the past two years, we have used this system to acquire volumetric scans from more than 180 subjects, with healthy and diseased retinas or optic nerve heads. Due to involuntary eye motion and reduced (or distorted) intensity of some OCT images, not all of the 3D scans are appropriate for volumetric reconstruction; the main factors being (especially among elderly patients) advanced cataract, significant eye aberrations, long eye lashes and ptosis.

We have tested the SVM segmentation on a variety of retinal structures. The amount of time required by the SVM segmentation methods depends on the complexity of the feature being isolated and the size of the data volume. The SVM runs more slowly when more training points (green and red painted regions) are specified. We found that, on a PC workstation having two Intel Xeon 3.6 GHz processors and 2 GB of main memory, the time to apply the SVM segmentation can be as short as a few minutes for small volumes with well-defined features and as long as a two hours for large volumes with complex structures. In addition, to evaluate the accuracy of the SVM-based segmentation, we have included a manual segmentation method that allows the specification of top and bottom polylines on individual B-scans for the purpose of isolating individual retinal layers. This manual specification is time-consuming, approximately requiring two hours per retinal layer, but it provides an accurate baseline for comparing SVM segmentations.

## Conclusion

Display technology that supports high-resolution visualization of massive image data is increasingly important for biomedical and life science applications. Our system enables scientists to control interactively a foveal inset display, which supports exploring selected areas of an image at a (physically) higher resolution than the surrounding display. The system can be constructed at lower cost than tiled displays with uniform resolution.

We use flow-based analysis to hierarchically segment a protein surface into protrusions and cavities. Our approach applies to any scalar function defined on the surface. We have used a geodesic distance-based alignment function in order to identify non-rigid sections of a protein [17]. We are now designing functions that capture various shape characteristics of the protein surface and plan to use the corresponding peak-valley segmentation in shape complementarity studies.

We found that our system works well at isolating most retinal layers and structures. We added speed improvements that also improved the system's tolerance to noisy data, something that is inherent in OCT data. These improvements are described in detail in [26]. The main problem we encountered was segmenting thin layers, on the order of one or two voxels in width. Some retinal layers are very thin, and we found that our system required more than expected user specification in order to isolate these regions. We are investigating improved segmentation methods and will possibly explore alternative artificial intelligence approaches to drive the segmentation process, but we expect the problem is rooted in the layers only being two voxels thick. Thus, the best solution is to increase the acquisition resolution to better resolve fine structures.

## Methods

### High-resolution display technology for exploration of biomedical image data

#### Hardware setup

Our tiled display wall consists of six tiles arranged in a three-by-two grid. Each tile has an area of 6' × 412′
 MathType@MTEF@5@5@+=feaafiart1ev1aaatCvAUfKttLearuWrP9MDH5MBPbIqV92AaeXatLxBI9gBaebbnrfifHhDYfgasaacH8akY=wiFfYdH8Gipec8Eeeu0xXdbba9frFj0=OqFfea0dXdd9vqai=hGuQ8kuc9pgc9s8qqaq=dirpe0xb9q8qiLsFr0=vr0=vr0dc8meaabaqaciaacaGaaeqabaqabeGadaaakeaacqaI0aandaWcbaWcbaGaeGymaedabaGaeGOmaidaaOWaaWbaaSqabeaakiadacTHYaIOaaaaaa@32B9@ for a total size of 18' × 9'. A tile is displayed using two Sanyo PLC-XT16 projectors to support stereoscopic imaging (note that our foveal inset system currently does not support stereoscopic rendring). The projectors are driven by a cluster of Linux machines with 2 GHz AMD Opteron processors and 1 GB of memory. The head node of the cluster is a Linux machine with dual 2 GHz AMD Opteron processors and 8 GB of memory. We are using Point Grey Flea [27] cameras for calibration and interaction. These cameras are capable of capturing 1024 × 768 pixel color images at 30 frames per second. We are using a Directed Perception Pan-Tilt Unit PTU-C46 to control the inset position, which can be moved at increments of 184 arc-seconds. Our unit is configured to move at 1000 incremental positions per second. We have mounted a mirror to the PTU using a gimbal adapter. The projector used to project the foveal inset is a Mitsubishi XD50U.

#### Software design

The position of the foveal inset is specified using a hand-held laser pointer. The control of the foveal inset position is implemented to work with a laser pointer interaction system [16]. The foveal inset controller receives information regarding the laser pointer position from the tracking application via a network socket. The foveal inset is positioned about this location on the display by adjusting the pan and tilt angles of the PTU. This allows the user to run-time specify the position of the foveal inset on the display, letting areas of interest be displayed in higher resolution than the rest of the display.

#### Integration

**Figure 9:**
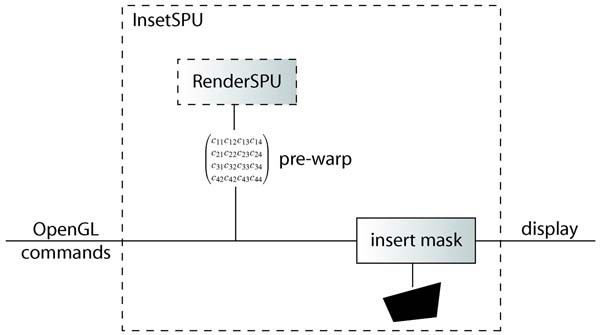
**Inset stream processing unit**. Illustration of InsetSPU used for rendering foveal inset with Chromium.

In order to modify the OpenGL pipeline without modifying application code, we have implemented the inset controller as a Chromium Stream Processing Unit (SPU) [28]. This SPU is a combination of the Chromium RenderSPU and PassthroughSPU. Our SPU derived from the RenderSPU, and also implements PassthroughSPU functionality. This allows it to render the inset locally and pass rendering information down the pipeline. The integration of the InsetSPU is depicted in Figure 9.

### Analysis and visualization of protein surfaces

**Figure 10:**
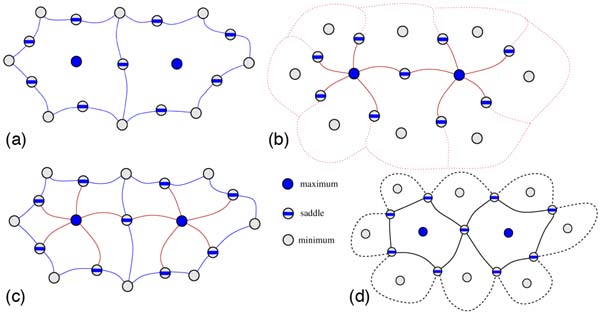
**Morse-Smale complex**. a: Descending manifolds of maxima and saddles. b: Ascending manifolds of minima and saddles. c: The Morse-Smale complex is obtained as an overlay of these ascending and descending manifolds. d: Paths connecting saddles within each cell of the MS complex segment the surface into peaks and valleys.

The segmentation shown in Figure 4 is derived from the *Morse-Smale complex *(MS complex), which partitions the surface into monotonic regions [18,29]. The critical points of the atomic density function, in particular the maxima and minima, have unique regions associated with them called *descending and ascending manifolds*. Using the analogy of terrains, where the scalar function describes the elevation of a point, maxima and minima correspond to "mountain peaks" and lowest points of "valleys." The descending manifold of a maximum *m *consists of all points *p *that satisfy the following property: the steepest-ascent path beginning at *p *terminates at *m*. Similarly, steepest-descent paths beginning at all points associated with a minimum terminate at the minimum (see Figure 10). Ascending and descending manifolds of saddles are one-dimensional curves on the surface.

The overlay of the descending and ascending manifolds results in a partition of the surface into monotonic regions. A cell in the partition consists of points that flow towards a fixed minimum/maximum pair via paths of steepest descent/ascent. The MS complex is a topological data structure that stores this segmentation into monotonic regions. Efficient algorithms have been developed to compute the MS complex and to generate representations of the function at multiple levels of detail [29,30]. These algorithms repeatedly cancel a pair of critical points connected by an arc in the MS complex to obtain a smoother function.

Given a protein surface and the atomic density function, we first construct the MS complex and then compute paths connecting saddles within each cell of the complex. These paths collectively form boundaries of peaks and valleys of the surface, see Figure 10. Peaks and valleys of the atomic density function correspond to protrusions and cavities of the protein thereby resulting in the required natural segmentation.

### Visualization and analysis of retinal optical coherence tomography

Recent developments in ultra-high-resolution Fourier domain optical coherence technology (Fd-OCT) allow the rapid collection of B-scan stacks such that alignment can be achieved by standard image registration methods. We have developed an Fd-OCT system for acquiring and processing image stacks. A detailed description of our hardware setup in provided in [26,31].

Each subject was imaged with several OCT scanning procedures including 3D scanning patterns centered at the fovea and optic nerve head (ONH). We used two different scanning arrangements for 3D scanning patterns including (i) regularly spaced 200 B-scans, with each based on 500 A-scans, and (ii) regularly spaced 100 B-scans based on 1000 A-scans. In both cases, volumes consisted of the same number of A-scans (100000), and the time required to acquire a volume was 5.5 *s *for 50 *μs *CCD exposure time and 11.1 *s *for 100 *μs *exposure time. The longer exposure time was mainly used to increase image intensity and with 100 B-scans based on 1000 A-scans per frame acquisition mode, at the cost of more motion artifacts.

## Competing interests

The authors declare that they have no competing interests.

## Authors' contributions

OS designed and supervised the development of the foveal inset display, and drafted the manuscript. VN developed the protein surface analysis and visualization system. GW developed the visualization tools for 3D gene expression. DW supervised the development of the algorithms, tools, and overall software for interactive visualization and analysis of retinal imaging data obtained from optical coherence tomography. BH helped to design and conceptualize the techniques discussed here. All authors contributed to writing and revising the manuscript, and read and approved the final manuscript.
